# Analysis of Single Nucleotide Variants (SNVs) Induced by Passages of Equine Influenza Virus H3N8 in Embryonated Chicken Eggs

**DOI:** 10.3390/v13081551

**Published:** 2021-08-05

**Authors:** Wojciech Rozek, Malgorzata Kwasnik, Wojciech Socha, Pawel Sztromwasser, Jerzy Rola

**Affiliations:** 1 Department of Virology, National Veterinary Research Institute, Al. Partyzantow 57, 24-100 Pulawy, Poland; malgorzata.kwasnik@piwet.pulawy.pl (M.K.); wojciech.socha@piwet.pulawy.pl (W.S.); jrola@piwet.pulawy.pl (J.R.); 2 Department of Omics Analyses, National Veterinary Research Institute, Al. Partyzantow 57, 24-100 Pulawy, Poland; pawel.sztromwasser@gmail.com

**Keywords:** equine influenza virus, vaccines, genetic variants, NGS, host adaptation, quasispecies

## Abstract

Vaccination is an effective method for the prevention of influenza virus infection. Many manufacturers use embryonated chicken eggs (ECE) for the propagation of vaccine strains. However, the adaptation of viral strains during subsequent passages can lead to additional virus evolution and lower effectiveness of the resulting vaccines. In our study, we analyzed the distribution of single nucleotide variants (SNVs) of equine influenza virus (EIV) during passaging in ECE. Viral RNA from passage 0 (nasal swabs), passage 2 and 5 was sequenced using next generation technology. In total, 50 SNVs with an occurrence frequency above 2% were observed, 29 of which resulted in amino acid changes. The highest variability was found in passage 2, with the most variable segment being IV encoding hemagglutinin (HA). Three variants, HA (W222G), PB2 (A377E) and PA (R531K), had clearly increased frequency with the subsequent passages, becoming dominant. None of the five nonsynonymous HA variants directly affected the major antigenic sites; however, S227P was previously reported to influence the antigenicity of EIV. Our results suggest that although host-specific adaptation was observed in low passages of EIV in ECE, it should not pose a significant risk to influenza vaccine efficacy.

## 1. Introduction

Influenza A virus (IAV) is a member of the *Orthomyxoviridae* family characterized by a wide range of hosts, including humans, birds, pigs, horses, marine mammals and others. Among the four types of influenza virus, IAV has the highest rate of mutations and causes the most severe diseases [1]. The genome of IAV is composed of eight segments of single-stranded, negative-sense RNA, packed in separate viral ribonucleoprotein complexes. The eight RNA segments (I-VIII) encode at least 10 proteins: polymerase basic 2 (PB2) (segment I), polymerase basic 1 (PB1) (segment II), polymerase acidic (PA) (segment III), hemagglutinin (HA) (segment IV), nucleoprotein (NP) (segment V), neuraminidase (NA) (segment VI), matrix protein 1 (M1) and 2 (M2) (segment VII), non-structural protein 1 (NS1) and 2 (NS2/NEP) (segment VIII) [2]. More recently, additional proteins that are encoded by the IAV genome have been identified: PB1-F2, PB1-N40, PA-X, PA-N155, PA-N182, M42 and NS3 [3,4,5,6,7]. HA and NA glycoproteins are the major determinants of antigenicity because they induce neutralizing antibody production. As with other RNA viruses, influenza exhibits high evolutionary dynamics through several molecular mechanisms including the genetic reassortment of segments, genetic recombination and point mutation, which often results in an antigenic shift or antigenic drift. An antigenic shift is a rapid change in the virus genome and antigenicity due to a reassortment of RNA segments of different viral strains producing new subtypes and in the case of influenza, causing recurring pandemics [8]. Antigenic drift is associated with point mutations occurring as a result of the low fidelity of viral RNA polymerases. As a consequence of their high mutation rate, influenza viruses exist as a large group of non-identical but closely related viral genomes called quasispecies, which are constantly competing and undergoing selection [9,10]. The high variability rate of IAV is a challenge for vaccination programs at both the vaccine design and formulation stages. It is well established that vaccine mismatch caused by the antigenic drift of the virus leads to the reoccurrence of influenza epidemics and creates the need for regular updates of the vaccine content [11]. A less studied aspect of the risk associated with genetic drift are the mutations induced during the manufacturing processes. Currently, most manufacturers use embryonated chicken eggs (ECE) for vaccine strain propagation, as this allows for the production of large amounts of high-titer viruses (https://www.cdc.gov/flu/prevent/how-fluvaccine-made.htm, accessed on 4 February 2021). Previous studies based on Sanger sequencing reported that the adaptation of influenza to chicken embryos may result in changes in hemagglutinin (HA) that could affect the receptor-binding specificity of the virus [12,13]. The goal of our studies was to analyze the distribution of the single nucleotide variants (SNVs) across the whole genome of the equine influenza virus (EIV) during low passages in ECE in order to evaluate viral variability resulting from host adaptation. The use of next generation sequencing (NGS) technology allowed us to determine of the relative frequency of SNVs within a quasispecies population.

## 2. Materials and Methods

### 2.1. Virus Propagation

Equine influenza virus A/equine/Pulawy/1/2005 (H3N8) was isolated in our laboratory and had been characterized previously [14]. The virus was propagated in 10-day-old embryonated specific pathogen-free (SPF) chicken eggs. Briefly, 0.2 mL of PCR-positive nasal swab solution was inoculated into the allantoic cavity of ECE. After 72 h of incubation at 37 °C, the amniotic-allantoic fluids were collected. Five consecutive passages were carried out, and the three fluids with the highest hemagglutination titers were combined and used for inoculation of 15 eggs or RNA extraction (p0, p2 and p5). Fluids showing incomplete hemagglutination (HA < 4) after the first passage were combined and used undiluted for the inoculation (p2). Fluids positive in p2 (HA ≥ 32) were combined and used for the next passage in 10^−2^ dilution in PBS, and for p4 and p5 (HA ≥ 128), the virus was diluted to 10^−3^. The amniotic-allantoic fluids from inoculated ECE were stored at −70 °C between passages.

### 2.2. Hemagglutination (HA) and Hemagglutination Inhibition (HI) Assays

The HA and HI assays were performed according to the OIE Manual (https://www.oie.int/fileadmin/Home/eng/Health_standards/tahm/3.06.07_EQ_INF.pdf; accessed on 11 February 2021). The HA titer was determined for all collected amniotic-allantoic fluids. Viruses obtained in p2 and p5 were tested in HI with the following reference sera: South Africa/4/03 (FL1 lineage), Newmarket/1/93 (American lineages) and Kentucky/81 (Pre-divergent).

### 2.3. Next Generation Sequencing and Data Analysis

Swab samples taken directly from a naturally infected horse and designated passage 0 (p0) and the amniotic-allantoic fluids from passage 2 (p2) and passage 5 (p5) were selected for further analysis. In order to compare the viral diversity generated at subsequent passages, samples were subjected to deep sequencing. Briefly, RNA extraction was performed using the RNeasy mini kit (Qiagen, Hilden, Germany) according to the manufacturer’s protocol. Each sample was treated with DNAse and reverse transcribed with the 5′AGCAAAAGCAGG′3 UNI12 primer [15] using Transcriptor Reverse Transcriptase (Roche, Basel, Switzerland). Viral RNA was sequenced on the Illumina MiSeq platform using a 2 × 250 read length with a MiSeq Reagent Kit v3 (Illumina, San Diego, CA, USA). The adapters were removed by merging overlapping read-pairs using bbmerge (https://jgi.doe.gov/data-and-tools/bbtools/; accessed on 19 December 2019) and trimming non-overlapping read-pairs with Trimmomatic [16]. Contigs were assembled using SPAdes 3.9.0 [17]. Reads were mapped to the A/equine/Richmond/1/2007 (H3N8) reference genome using the Burrows–Wheeler alignment BWA MEM algorithm [18]. The coverage depth for each base and segment was calculated using Samtools software (Galaxy version 1.9) with no depth limit set and MQ > 0 [19]. Variant calling was performed using Freebayes [20]. Single nucleotide variants with a frequency greater than 2% in any virus passage were considered.

In order to assess the diversity of the virus populations, Shannon entropy was calculated for each RNA segment separately and also for whole viral genomes. The following formula was used, where *f_i_* was the frequency of the nucleotide A, C, G or T at position i and N was the total length of the segment [21,22].

(1) $$\mathrm{SE}=\;-\frac{1}{N}\overset{N}{\underset{i=1}{\sum }}(f_{\mathrm{iA}}\;\ln f_{\mathrm{iA}}\;+f_{\mathrm{iG}}\ln f_{\mathrm{iG}}\;+f_{\mathrm{iT}}\ln f_{\mathrm{iT}}+f_{\mathrm{iC}}\;\ln f_{\mathrm{iC}})$$

## 3. Results

Full-length nucleotide sequences of all influenza virus RNA segments were obtained from nasal swabs of the naturally infected horse (p0) and amniotic-allantoic fluid samples (p2 and p5). The average coverage depths were 948.54 for p0, 7447.38 for p2 and 15,013.54 for p5 (Table S1). The sequences were submitted to Genbank: p0 (MZ373289-MZ373296), p2 (MZ364009-MZ364016) and p5 (MZ364001-MZ364008).

**Figure 1:**
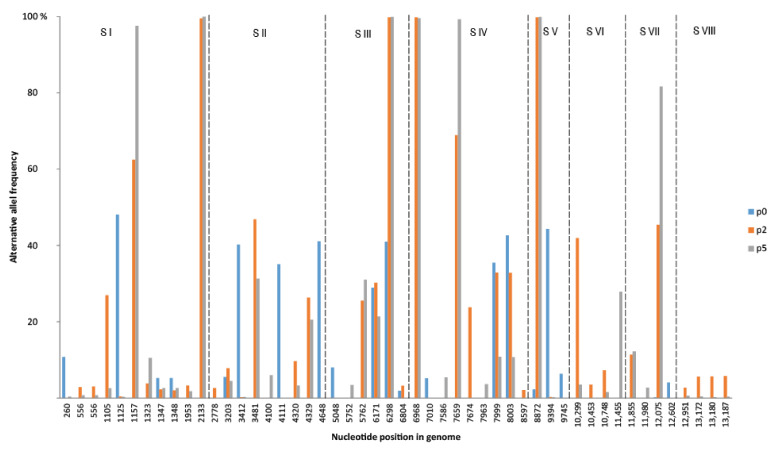
The frequency and distribution of single nucleotide variants of the A/equine/Pulawy/2005 detected in nasal swabs (passage 0) and passages in embryonated chicken eggs (passage 2 and passage 5). S I–S VIII genome segments.

NGS sequencing data analysis allowed for the identification of 50 SNVs within eight segments of the influenza virus genome. Among them, 29 induced a change in an amino acid, 20 were synonymous, and 1 variant was in the non-coding region of the segment. The positions with variations were distributed as follows: segment I divulged 5 amino acid changes in 11 variants (I—5/11), segment II—6/9, segment III—4/6, segment IV—5/9, segment V—1/3, segment VI—3/4, segment VII—3/4, and segment VIII—2/4 (Table 1, Figure 1). A permanent change in the dominant amino acid variant was observed between p0 and p5 at three positions: segment I (PB2 (A377E)), segment III (PA (R531K)) and segment IV (HA (W222G)).

**Figure 2:**
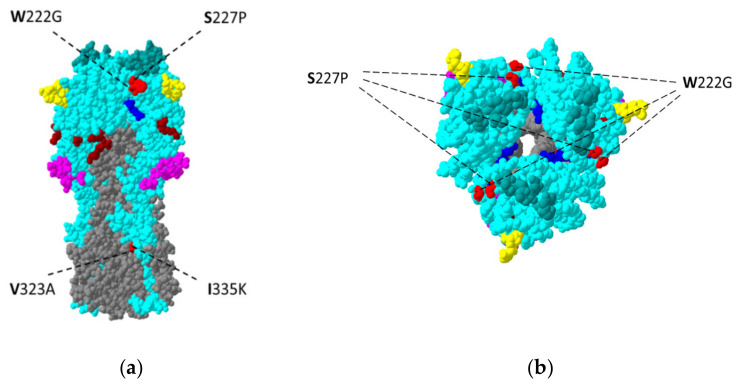
Single nucleotide variants (red) detected in the hemagglutinin protein of the A/equine/Pulawy/1/2005 (H3N8) strain during passages in embryonated chicken eggs. Regions of hemagglutinin are distinguished by color: teal—HA1; gray—HA2; as are five antigenic sites: yellow—antigenic site A; green—antigenic site B; pink—antigenic site C; dark blue—antigenic site D; and brown—antigenic site E. (**a**) Side view; (**b**) top view. The three-dimensional model is based on the crystal structure of A/equine/Richmond/1/2007 (H3N8) HA (PDB accession no. 4UO0) and visualized using Swiss-PdbViewer 4.1.0. [25].

Four of the five amino acid variants—W222G, S227P, V323A and I335K—observed in segment IV (HA) are depicted in Figure 2. None of them were located within any of the previously defined major antigenic regions: A (aa 140–146), B (aa 155–160/187–196), C (aa 44–54/275–280), D (aa 207–212), E (aa 62–64/83/260–264). However, two of them, W222G and S227P, were located within the *loop220* domain [23,24].

**Figure 3:**
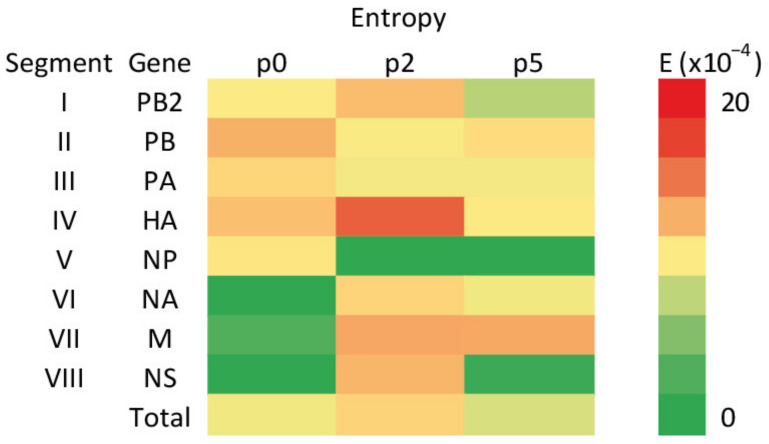
Heatmap of Shannon entropy for EIV genome segments and whole genomes, calculated from the frequencies of variants present in the virus population in nasal swabs (p0), passage 2 (p2) and passage 5 (p5).

In order to characterize the differences in the complexity of the viral population, the per-site Shannon entropy was calculated, considering the frequencies of nucleotide substitutions across each of the genome segments, and visualized with a heatmap (Figure 3, Table S2).

Fluctuations of entropy measured between passages that were observed for each of the segments with the overall genome complexity peaking in p2. The highest values were observed for segment IV (9.7 × 10^−4^ on average) with the peak of complexity occurring in p2 (14.3 × 10^−4^). Additionally, in segments I, VI and VIII, p2 was the passage in which the highest entropy was observed. In contrast, complexity was reduced to 0 for segment V starting from p2.

Viruses obtained in p2 and p5 were tested in HI with the reference sera previously referred to. There was no difference in p2 and p5 reactivity: HI 1/32 for South Africa/4/03 -, HI 1/32 for Newmarket/1/93 and HI 1/256 for Kentucky/81. The virus from p0 showed HA < 4, and therefore the HI assay was not feasible.

## 4. Discussion

Vaccination is an effective method used for the prevention of influenza virus infections. The efficacy of the vaccine may be reduced as a result of antigenic differences between the vaccine and the field virus strains. The antigenic evolution of EIV is closely monitored by OIE experts and the genetic background of these changes is studied. In addition to the genetic variability of the virus in the population, mutations induced during vaccine production may also compromise vaccine efficacy. The use of ECE for the multiplication of mammalian influenza viruses may engender the risk of changes related to adaptation to a new host, since influenza viruses propagated with this method have been known to undergo phenotypic changes affecting their pathogenic profile since the 1940s [26]. Said et al. (2011) used Sanger sequencing to identify amino acid positions substituted upon adaptation of EIV H3N8 to the chicken host [27]. However, in the case of the influenza virus, it seems to be more reliable to analyze the viral quasispecies populations rather than focus on consensus sequences based on dominant variants. This has become possible with the application of NGS technology. By using this method, we studied the effect of low passages of EIV in ECE in the context of viral quasispecies. It can be assumed that the viral heterogeneity observed at passage 0 reflected the primary host–virus equilibrium, whereas the variability observed in consecutive passages involved changes associated with the adaptation to ECE. In the study by Said et al. (2011), six amino acid substitutions were identified in the course of multiple passages of EIV [27]. Those changes were located not only in the highly variable surface glycoprotein HA, but also in the internal proteins M1, NP and PA. The next generation sequencing performed in our study identified variable positions in each segment of the viral genome. The Shannon entropy calculations for genetic sequences of virus from p0, p2 and p5 gave a measure of viral genetic diversity. The highest average entropy for the whole genome was found in p2, whereas the lowest was observed in p5. The most diverse segment was IV (HA). In segments I (PB2), IV (HA), VI (NA), VII (M) and VIII (NS), entropy increased in p2, then decreased or remained constant in p5. The change in host was accompanied by an increase in the viral variability in p2, while a stabilization and a decrease were observable in p5. A similar tendency was found in a previous study, when changing the host from turkeys to chickens led to an early increase in the overall genetic variability of influenza virus strains followed by a decrease in this indicator by the fifth passage [28].

Out of the 50 identified SNVs, 29 resulted in changes in amino acids. Among non-synonymous SNVs, only 14 occurred with frequencies over 10%. Hemagglutinin and neuraminidase (NA) proteins are the major targets of neutralizing antibodies induced by infection or vaccination and determinants of pathogenicity and host specificity. The former protein binds to sialoglycoprotein on the surface of the host cell and mediates virus attachment, and the latter removes sialic acid from viral receptors and allows the release of progeny virus from host cells [29]. Adaptation to novel host species by the influenza virus requires the readjustment of the functional balance of the sialic acid receptor–binding HA and the receptor-destroying NA to the sialoglycan receptor of the new host [30]. Mutations affecting antigenicity and associated with adaptation investigated in eggs have been identified within HA of the human H3N2 influenza virus strains H156Q [31], L194P [32], and T160K [33]. In our analysis, we found five positions with amino acid substitutions within the HA sequence: W222G, S227P, V323A, I335K and F534L (Table 1). All of these changes were located outside of the major antigenic sites of HA. The study of the antigenic characterization of viruses obtained during passaging would require the preparation of specific cross-reactive sera against p0, p2 and p5 viruses. To address the issue to some extent, we tested p2 and p5 viruses by HI using three reference sera. There were no differences in the reactivity of p2 and p5 viruses, which seems to be consistent with our SNV analysis showing no variants to be induced strictly at the major antigenic sites of HA. This supports our assumption that low passages in ECE should not pose a significant risk to vaccine production.

Substitutions in HA at positions V323A and F534L appeared only in p2 or p5 and did so with very low frequencies (3.63 and 2.08%). The change at I335K within the fusion peptide of HA2 occurred in p0, p2 and p5 with a frequency above 10% but remained a minority variant. Changes at residues 222 and 227 seemed to be important, as these residues are both located within the receptor-binding site *loop220* of hemagglutinin [23]. In our experiment, the W222G substitution became more fixed in subsequent passages, starting from 0% in p0 but reaching 99.25% in p5. This position has been postulated as affecting the adaptation to the new host [27]. Casalegno et al. (2014) confirmed that changes at position 222 affect the intensity of binding for SAa-2,6 and alter the HA–NA balance, which may influence viral fitness [34]. Mutation W222L could facilitate the viral adaptation of EIV H3N8 to dogs [35]. Yang et al. (2019) described finding predominately tryptophan (W) at residue 222 in the HA of avian H3N2, whereas they observed leucine (L) at this position in canine IAV [12]. The W222G substitution observed in our study seemed to be linked to the propagation of the virus in ECE as it became dominant. Other studies have also reported that the mutation at residue 222 in HA can affect influenza viral infectivity in mice [36]. The same substitution was reported by Ilobi et al. (1994) as being associated with the adaptation of equine H3N8 to Madin–Darby canine kidney cells [37]. The S227P substitution appeared in p2 (where the frequency was 23.73%) but was not observed in p5. Woodward et al. (2015) postulated that mutation at this position may alter not only receptor-binding activity, but also the antigenicity of the virus [38]. This position is also speculated to be critical for the HI reactivity induced by H5 influenza vaccines. However, whether the effect of residue 227 on immunogenicity can be generalized for different subtypes is unclear [39]. In a study analyzing H5N2 AIV strains, Hiono et al. (2016) proved that the glycan-binding specificity of HA was determined by amino acid residues at positions 222 and 227, together with NA [40]. However, in our study, among three nonsynonymous mutations within NA—G11R, I62N, and N396S—none were directly involved in substrate binding.

Proteins of the influenza virus polymerase complex (PB2, PB1 and PA) have been postulated to be involved in the adaptation of avian IAVs to mammalian hosts [41]. The PB2 protein plays a role in viral transcription and has been found to affect the host range as well as the virulence of the influenza viruses [42,43,44]. We observed that the A377E alteration appeared in p2 (62.45%), and its frequency had increased in p5 (97.54%). This substitution is located in the cap-binding domain (PB2_319–481_) and may be important in the context of mammalian/avian adaptation. The region of PB2 between amino acids 362 and 581 is believed to promote replication in mammalian cells, and single amino acid changes in this sequence contribute to increased virulence in mice [45]. The PA subunit of avian influenza virus may play a major role in the host adaptation. The pandemic H1N1pdm09 virus acquired multiple mutations in the PA gene that promotes polymerase activity in mammalian cells, even in the absence of previously identified host adaptive mutations in other polymerase genes [46]. The substitution in PA observed in our experiment, which is R531K, is located within the large PA-C domain, and this binds the 15 first residues of the PB1 subunit, which is also involved in viral mRNA transcription [47]. The frequency of the substituted R531K variant increased from 40.98% in p0 to over 99% in p2 and p5.

It was speculated that the M1 protein may be involved in host tropism [48]. We found one M1 SNV—A41V, with the rate of the alternative variant exceeding 10% of that which appeared in p2. This mutation was previously described as being associated with a morphological change in the shape of influenza virus particles from a predominantly filamentous to spherical form. It was conjectured that the filamentous form could present some advantages for the virus in a natural host [49].

## 5. Conclusions

Our results have shown that the passaging of EIV in ECE affects the composition of quasispecies populations. Genetic variants were detected in all EIV segments, and this may reflect the mechanisms of host adaptation. In general, variability was found to be higher in passage 2 than in passage 5, with the most variable being segment IV, encoding hemagglutinin (HA). In most cases, alternative variants appeared with low frequency or did not alter amino acid composition. The frequency of three variants—HA (W222G), PB2 (A377E) and PA (R531K)—clearly increased with the number of passages, becoming dominant. None of the SNVs in the HA gene were located within the regions encoding major antigenic sites; however, variant S227P may affect viral antigenicity. In summary, our results suggest that although host specific adaptation was observed in low passages of EIV in ECE, it seems unlikely that it could create a risk for influenza vaccines’ efficacy.

## Figures and Tables

**Table 1:** Single nucleotide variants of equine influenza virus H3N8 strain A/equine/Pulawy/2005 passaged in embryonated chicken eggs.

Segment (Gene)	Nucleotide			Amino Acid			Passage 0		Passage 2		Passage 5	
	Position	Ref ^1^	Alt ^2^	Position	Ref	Alt	Alt (%)	Ref/Alt	Alt (%)	Ref/Alt	Alt (%)	Ref/Alt
I (PB2)	260	G	T	78	W	L	10.78	662/80	0.00		0.41	8699/36
	556	C	T	177	I	syn ^3^	0.00		2.89	6289/187	0.71	6613/47
	556	A	C	177	I	L	0.00		3.01	6281/195	0.72	6612/48
	1105	T	G	360	Y	D	0.00		26.93	2936/1082	2.52	3668/95
	1125	C	T	366	V	syn	48.02	184/170	0.43	3919/17	0.30	3634/11
	1157	C	A	377	A	E	0.00		62.45	1458/2452	97.54	83/3297
	1323	T	C	432	H	syn	0.00		3.79	2790/110	10.48	2075/243
	1347	G	A	440	K	syn	5.26	252/14	2.29	2641/62	2.62	2084/56
	1348	A	G	441	N	D	5.26	252/14	2.00	2640/54	2.60	2063/55
	1953	G	A	642	G	syn	0.00		3.30	1406/48	1.79	1375/25
	2133	G	A	702	K	syn	0.00		99.44	9/1611	99.96	2/4884
II (PB1)	437	C	T	138	P	L	0.00		2.62	7832/211	0.00	
	862	G	A	280	A	T	5.51	343/20	7.80	4147/351	4.48	7402/347
	1071	G	A	349	A	syn	40.20	180/121	0.24	2919/7	0.27	4808/13
	1140	G	A	372	M	I	0.00		46.87	1521/1342	31.29	3261/1485
	1759	C	A	579	L	M	0.00		0.00		5.98	2264/144
	1770	G	A	582	Q	syn	35.05	126/68	0.00		0.00	
	1979	C	G	652	A	G	0.00		9.67	1729/185	3.31	1780/61
	1988	T	G	655	M	R	0.00		26.30	1864/665	20.54	2100/543
	2307	G	A	-	non coding	-	41.05	56/39	0.00		0.00	
III (PA/PA-X)	366	G	A	114	E	syn	8.01		0.00		0.00	
(PA)	1070	A	G	349	E	G	0.00		0.00		3.44	5748/205
	1080	A	G	352	E	syn	0.00		25.53	4805/1647	31.00	3888/1747
	1489	A	T	489	S	C	28.92	644/262	30.21	7470/3234	21.38	11,306/3074
	1616	G	A	531	R	K	40.98	743/516	99.80	26/12,685	99.89	23/21,153
	2122	G	C	700	V	L	1.88	313/6	3.21	5546/184	0.00	
IV (HA-signal)	47	T	C	6	I	syn	0.00		99.76	20/8254	99.54	75/16,404
(HA1)	89	C	A	5	I	syn	5.20	1731/95	0.00		0.00	
	665	A	G	197	Q	syn	0.00		0.00		5.42	10,604/608
	738	T	G	222	W	G	0.00		68.88	1812/4010	99.25	73/9723
	753	T	C	227	S	P	0.00		23.73	4422/1376	0.00	
	1042	T	C	323	V	A	0.00		0.00		3.63	9924/374
(HA2)	1078	T	A	335	I	K	35.47	464/255	32.84	9714/4749	10.84	9728/1183
	1082	G	A	336	A	syn	42.65	464/345	32.81	9853/4812	10.73	9755/1172
	1676	T	A	534	F	L	0.00		2.08	3299/70	0.00	
V (NP)	189	A	G	48	K	syn	2.33	882/21	99.79	20/9610	99.87	19/14,348
	711	T	G	222	I	M	44.32	250/199	0.26	4953/13	0.14	8296/12
	1062	G	A	339	E	syn	6.33	296/20	0.00		0.00	
VI (NA)	51	G	A	11	G	R	0.00		41.89	4702/3389	3.52	13,578/496
	205	T	A	62	I	N	0.00		3.57	8696/322	0.00	
	500	A	G	160	K	syn	0.00		7.30	7501/591	1.56	13,249/210
	1207	A	G	396	N	S	0.00		0.00		27.88	6035/2333
VII (M1)	147	C	T	41	A	V	0.00		11.42	14,515/1871	12.23	25,778/3593
	272	G	A	83	A	T	0.00		0.00		2.71	16,057/448
	367	A	G	114	E	syn	0.15	646/1	45.42	7291/6067	81.64	3202/14,237
(M2)	894	A	G	61	R	G	4.06	307/13	0.00		0.00	
VIII (NS1)	216	A	G	64	I	V	0.00		2.73	12,270/344	0.66	19,306/129
	437	C	T	137	I	syn	0.00		5.59	9605/569	0.43	17447/76
	445	A	G	140	K	R	0.00		5.66	9393/564	0.22	17,413/38
	452	G	A	142	E	syn	0.00		5.78	9184/563	0.40	16,970/68

^1^ Dominant variant in passage 0, A/equine/Pulawy/1/2005; ^2^ Alternative variant; ^3^ Synonymous variant.

## Data Availability

Data supporting the reported results can be requested from the corresponding author.

## Supplementary Materials

The following are available online at https://www.mdpi.com/article/10.3390/v13081551/s1, Table S1: Quantitation of equine influenza virus specific reads and sequence coverage; Table S2: Shannon entropy calculation.
