# Supplementary material for: Analysis of Single Nucleotide Variants (SNVs) Induced by Passages of Equine Influenza Virus H3N8 in Embryonated Chicken Eggs
Source: Viruses. 2021 Aug 5;13(8):1551. doi: 10.3390/v13081551 (PMC8402691; doi:10.3390/v13081551)
Supplement: Supplementary file 1 [file viruses-13-01551-s001.zip › viruses-1289431-supplementary.pdf]

viruses

MDPI

Supplementary Materials

Analysis of Single Nucleotide Variants (SNVs) Induced by Passages of Equine Influenza Virus H3N8 in Embryonated Chicken Eggs

Wojciech Rozek [1,*], Malgorzata Kwasnik [1], Wojciech Socha [1], Pawel Sztromwasser [2] and Jerzy Rola [1]

[1] Department of Virology, National Veterinary Research Institute, Al. Partyzantow 57, 24-100 Pulawy, Poland; malgorzata.kwasnik@piwet.pulawy.pl (M.K.); wojciech.socha@piwet.pulawy.pl (W.S.); jrola@piwet.pulawy.pl (J.R.)
[2] Department of Omics Analyses, National Veterinary Research Institute, Al. Partyzantow 57, 24-100 Pulawy, Poland; pawel.sztromwasser@gmail.com
* Correspondence: wojciech.rozek@piwet.pulawy.pl

Citation: Rozek, W.; Kwasnik, M.; Socha, W.; Sztromwasser, P.; Rola, J. Analysis of Single Nucleotide Variants (SNVs) Induced by Passages of Equine Influenza Virus H3N8 in Embryonated Chicken Eggs. *Viruses* **2021**, *13*, 1551. https://doi.org/10.3390/v13081551

Academic Editor: Romain Paillot

Received: 22 June 2021
Accepted: 2 August 2021
Published: 5 August 2021

Publisher's Note: MDPI stays neutral with regard to jurisdictional claims in published maps and institutional affiliations.

Copyright: © 2021 by the authors. Licensee MDPI, Basel, Switzerland. This article is an open access article distributed under the terms and conditions of the Creative Commons Attribution (CC BY) license (http://creativecommons.org/licenses/by/4.0/).

Table S1. Quantitation of equine influenza virus specific reads and sequence coverage.

Passage	Gene Segment	Coverage (%)	Mean Depth of Coverage	SD[1]
p0				
	I	99.62	717.40	548.56
	II	99.53	663.79	492.23
	III	100.00	1177.75	735.39
	IV	99.38	1040.59	516.22
	V	99.30	921.27	668.89
	VI	99.79	952.39	549.81
	VII	99.61	1180.81	592.67
	VIII	99.89	1321.16	848.44
	Genome	99.62	948.54	647.97
p2				
	I	100.00	4665.27	3282.33
	II	100.00	4683.51	3003.15
	III	100.00	8838.79	3103.51
	IV	100.00	9684.89	3554.06
	V	100.00	5847.30	2712.28
	VI	100.00	8468.62	2824.05
	VII	100.00	12460.62	3708.34
	VIII	100.00	9452.70	3584.32
	Genome	100.00	7447.38	4045.48
p5				
	I	99.49	7437.87	5727.04
	II	99.53	9906.11	7426.64
	III	98.16	16176.94	7279.86
	IV	98.64	17231.17	6241.76
	V	99.04	12918.27	5191.05
	VI	99.25	19016.68	5779.81
	VII	100.00	26473.59	7372.68
	VIII	99.10	24943.36	7102.19
	Genome	99.10	15013.54	8756.94

[1]Standard deviation of coverage depth.

Table S2. Shannon entropy calculation.

**Segment (Gene)**			**Passage 0**						**Passage 2**						**Passage 5**					
	**Nucleotide Position**		**Nucleotide Frequency**				**Shannon Entropy**		**Nucleotide Frequency**				**Shannon Entropy**		**Nucleotide Frequency**				**Shannon Entropy**	
	**Genome**	**Segment**	**A**	**G**	**C**	**T**	**Position**	**Segment**	**A**	**G**	**C**	**T**	**Position**	**Segment**	**A**	**G**	**C**	**T**	**Position**	**Segment**
I (PB2)	260	260	0	0.89	0	0.11	0.346515		0	1	0	0	0		0	1	0	0	0	
	556	556	0	0	1	0	0		0.03	0	0.94	0.03	0.268556		0.01	0	0.98	0.01	0.111902	
	1105	1105	0	0	0	1	0		0	0.27	0	0.73	0.583259		0	0.03	0	0.97	0.134742	
	1125	1125	0	0	0.52	0.48	0.692347		0	0	1	0	0		0	0	1	0	0	
	1157	1157	0	0	1	0	0	**0.000613**	0.62	0	0.38	0	0.664064	**0.000885**	0.98	0	0.02	0	0.098039	**0.000443**
	1323	1323	0	0	0	1	0		0	0	0.04	0.96	0.167944		0	0	0.1	0.9	0.325083	
	1347	1347	0.05	0.95	0	0	0.198515		0.02	0.98	0	0	0.098039		0.03	0.97	0	0	0.134742	
	1348	1348	0.95	0.05	0	0	0.198515		0.98	0.02	0	0	0.098039		0.97	0.03	0	0	0.134742	
	1953	1953	0	1	0	0	0		0.03	0.97	0	0	0.134742		0.02	0.98	0	0	0.098039	
	2133	2133	0	1	0	0	0		0.99	0.01	0	0	0.056001		1	0	0	0	0	
II (PB1)	2778	437	0	0	1	0	0		0	0	0.97	0.03	0.134742		0	0	1	0	0	
	3203	862	0.06	0.94	0	0	0.226967		0.08	0.92	0	0	0.278769		0.04	0.96	0	0	0.167944	
	3412	1071	0.4	0.6	0	0	0.673011		0	1	0	0	0		0	1	0	0	0	
	3481	1140	0	1	0	0	0		0.47	0.53	0	0	0.691346		0.31	0.69	0	0	0.619101	
	4100	1759	0	0	1	0	0	**0.000950**	0	0	1	0	0	**0.000611**	0.06	0	0.94	0	0.226967	**0.000710**
	4111	1770	0.35	0.65	0	0	0.647447		0	1	0	0	0		0	1	0	0	0	
	4320	1979	0	0	1	0	0		0	0.1	0.9	0	0.32508		0	0.03	0.97	0	0.134742	
	4329	1988	0	0	0	1	0		0	0	0	1	0		0	0.21	0	0.79	0.513957	

	4648	2307	0.41	0.59	0	0	0.676858		0	1	0	0	0		0	1	0	0	0	
III (PA/PA-X)	5048	366	0.08	0.92	0	0	0.278769		0	1	0	0	0		0	1	0	0	0	
(PA)	5752	1070	1	0	0	0	0		1	0	0	0	0		0.97	0.03	0	0	0.134742	
	5762	1080	1	0	0	0	0		0.74	0.26	0	0	0.573057		0.59	0.31	0	0	0.674370	
	6171	1489	0.71	0	0	0.29	0.602151	**0.000742**	0.7	0	0	0.3	0.610864	**0.000591**	0.79	0	0	0.21	0.513958	**0.000592**
	6298	1616	0.41	0.59	0	0	0.676858		1	0	0	0	0		1	0	0	0	0	
	6804	2122	0	0.98	0.02	0	0.098039		0	0.97	0.03	0	0.134742		0	1	0	0	0	
IV (HA-signal)	6968	47	0	0	0	1	0		0	0	1	0	0		0	0	0	1	0	
(HA1)	7010	89	0.05	0	0.95	0	0.198515		0	0	1	0	0		0	0	1	0	0	
	7586	665	1	0	0	0	0		1	0	0	0	0		0.95	0.05	0	0	0.198515	
	7659	738	0	0	0	1	0		0	0.69	0	0.31	0.619101		0	0.99	0	0.01	0.056001	
	7674	753	0	0	0	1	0	**0.000865**	0	0	0.24	0.76	0.551080	**0.001435**	0	0	0	1	0	**0.000631**
	7963	1042	0	0	0	1	0		0	0	0	1	0		0	0	0.04	0.96	0.167944	
(HA2)	7999	1078	0.35	0	0	0.65	0.647447		0.33	0	0	0.67	0.634179		0.11	0	0	0.89	0.346515	
	8003	1082	0.43	0.57	0	0	0.683315		0.33	0.67	0	0	0.634179		0.11	0.89	0	0	0.346515	
	8597	1676	0	0	0	1	0		0.02	0	0	0.98	0.098039		0	0	0	1	0	
V (NP)	8872	189	0.98	0.02	0	0	0.098039		0	1	0	0	0		0	1	0	0	0	
	9394	711	0	0.44	0	0.56	0.685930	**0.000646**	0	0	0	1	0	**0**	0	0	0	1	0	**0**
	9745	1062	0.06	0.94	0	0	0.226967		0	1	0	0	0		0	1	0	0	0	
VI (NA)	10299	51	0	1	0	0	0		0.42	0.58	0	0	0.680292		0.04	0.96	0	0	0.167944	
	10453	205	0	0	0	1	0	**0**	0.04	0	0	0.96	0.167944	**0.000755**	0	0	0	1	0	**0.000588**

	10748	500	1	0	0	0	0		0.93	0.07	0	0	0.253639		0.98	0.02	0	0	0.098039	
	11455	1207	1	0	0	0	0		1	0	0	0	0		0.72	0.28	0	0	0.592953	
VII (M1)	11855	147	0	0	1	0	0		0	0	0.89	0.11	0.346515		0	0	0.88	0.12	0.366925	
	11980	272	0	1	0	0	0	**0.000164**	0	1	0	0	0	**0.001007**	0.03	0.97	0	0	0.134742	**0.000994**
	12075	367	1	0	0	0	0		0.55	0.45	0	0	0.688139		0.28	0.82	0	0	0.519160	
(M2)	12602	894	0.96	0.04	0	0	0.167944		1	0	0	0	0		1	0	0	0	0	
VIII (NS1)	12951	216	1	0	0	0	0		0.97	0.03	0	0	0.134742		0.99	0.01	0	0	0.056001	
	13172	437	0	0	1	0	0	**0**	0	0	0.94	0.06	0.226967	**0.000916**	0	0	1	0	0	**0.0000629**
	13180	445	1	0	0	0	0		0.94	0.06	0	0	0.226967		1	0	0	0	0	
	13187	452	0	1	0	0	0		0.06	0.94	0	0	0.226967		0	1	0	0	0	
I-VIII (Total)	1-13625							**0.000589**						**0.000757**						**0.000519**
